# Supplementary material for: Virulence of Pseudomonas aeruginosa in Cystic Fibrosis: Relationships between Normoxia and Anoxia Lifestyle
Source: Antibiotics (Basel). 2023 Dec 19;13(1):1. doi: 10.3390/antibiotics13010001 (PMC10812786; doi:10.3390/antibiotics13010001)
Supplement: Supplementary file 1 [file antibiotics-13-00001-s001.zip › antibiotics-2767080-supplementary.pdf]

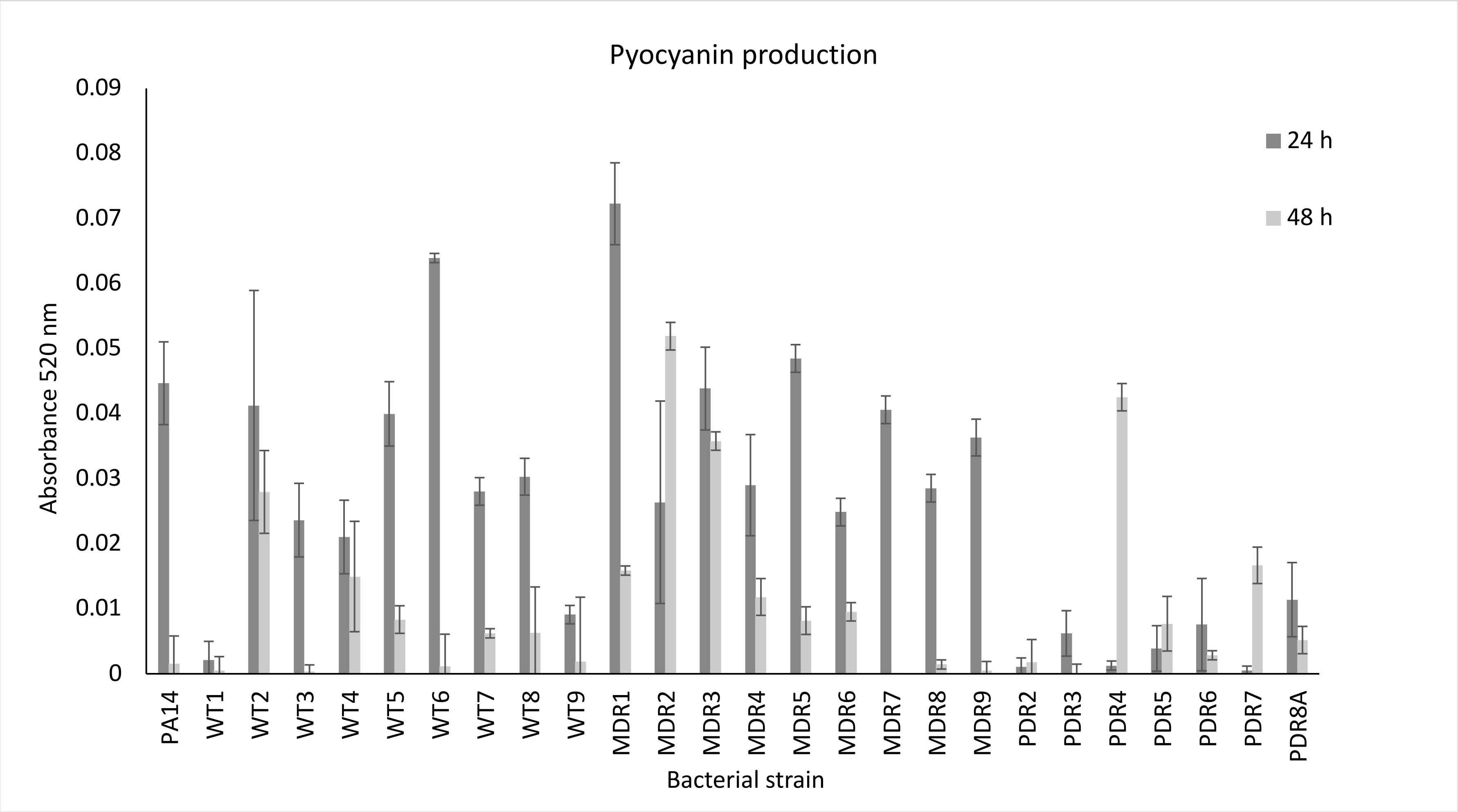


**Figure S1.** Pyocyanin production of *P. aeruginosa* clinical and reference strains. Pyocyanin production was evaluated after 24 h and 48 h of incubation at 37 °C in normoxia. The data are reported as OD at 520nm after pyocyanin extraction. Each data point represents the mean ± SD of 2 independent experiments, each performed in 3-replicates. Error bars indicate the standard deviations of all the measurements. WT: wild type, sensitive strains; MDR: multi-drug resistant strains; PDR: pan-drug resistant strains.

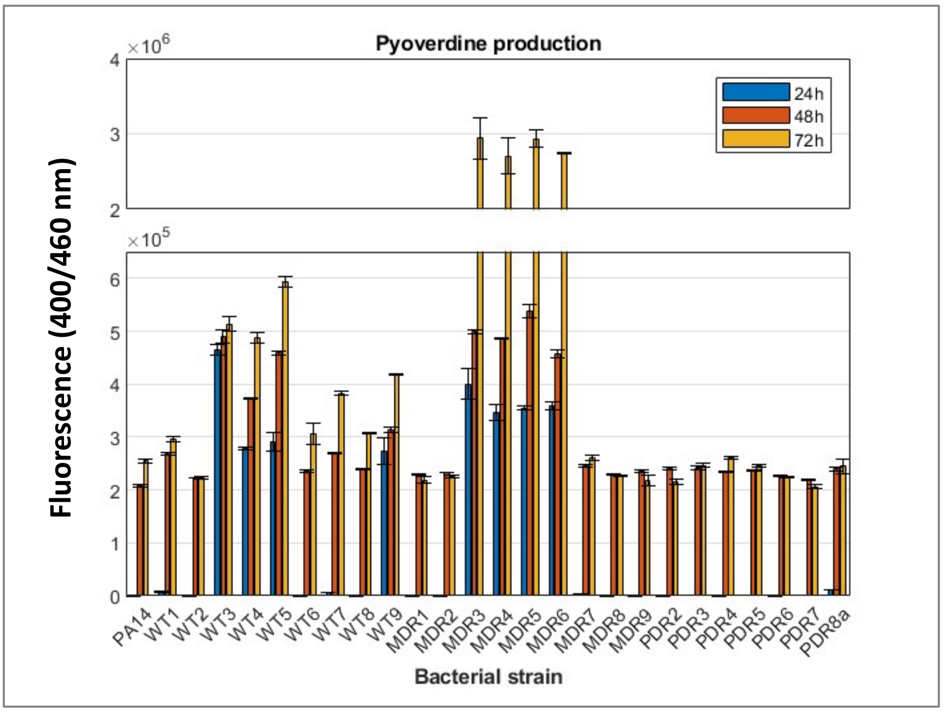


**Figure S2.** Pyoverdine production of WT, MDR and PDR strains at 24 h, 48 h and 72 h in normoxia. WT: wild type, sensitive strains; MDR: multi-drug resistant strains; PDR: pan-drug resistant strains.

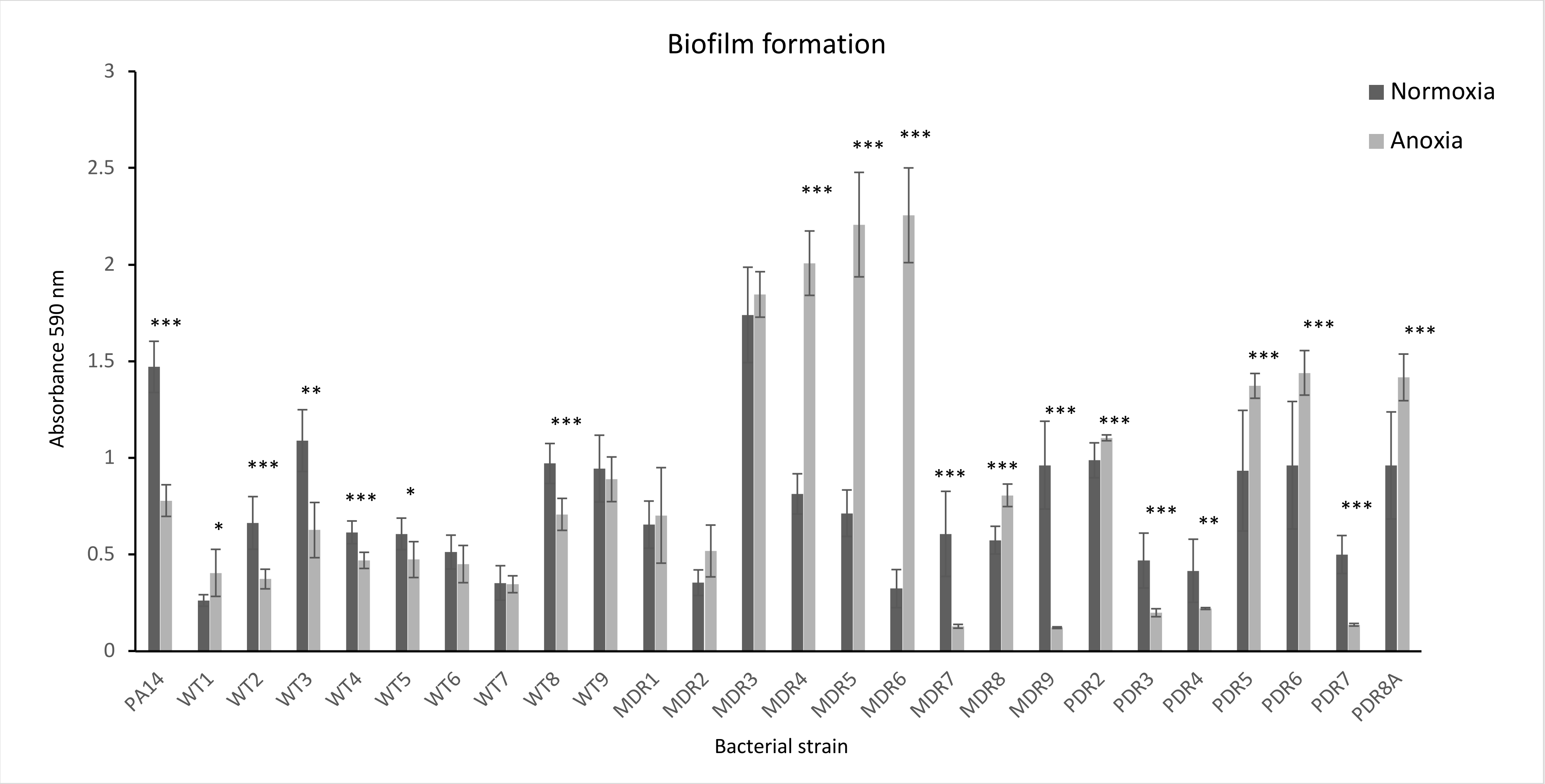


**Figure S3.** Biofilm formation of *P. aeruginosa* clinical and reference strains. Biofilm formation was evaluated after 18 h of incubation in polystyrene plates at 37 °C in normoxia and anoxia. Data are reported as OD at 590 nm after crystal violet staining. Each data point represents the mean ± SD of 4 independent experiments, each performed in 6-replicates. Error bars indicate the standard deviations of all the measurements. Statistical difference was determined by Student's T-test: * $p < 0.05$; ** $p < 0.01$,*** $p < 0.001$ compared with the control. WT: wild type, sensitive strains; MDR: multi-drug resistant strains; PDR: pan-drug resistant strains.

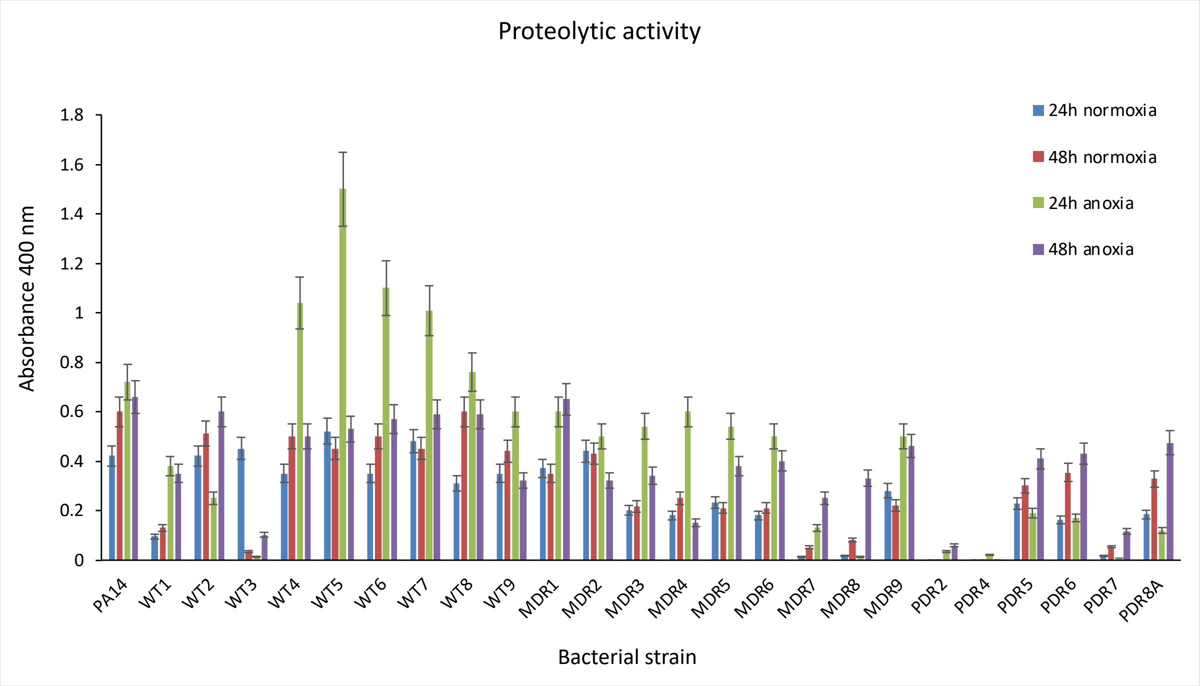


**Figure S4.** Protease production of different clinical strains and PA14 reference strain at 24 h and 48 h in normoxia and anoxia. Data are expressed as azocasein absorbance normalized for OD of each bacterial culture. Error bars indicated the standard deviations of all the measurements. WT: wild type, sensitive strains; MDR: multi-drug resistant strains; PDR: pan-drug resistant strains.

Table S1: Diameters of swimming motility of WT, MDR and PRD strains art 24, 48 and 72 h in normoxia and anoxia, respectively.

| **Normoxy** | 24h (cm) | 48h (cm) | 72h (cm) |
|---|---|---|---|
| PA14 | 2.3 ± 0.2 | 2.9 ± 0.2 | 3.2 ± 0.3 |
| WT1 | 2.9 ± 0.2 | 3.6 ± 0.3 | 4.2 ± 0.4 |
| WT2 | 3.2 ± 0.3 | 3.8 ± 0.4 | 4.5 ± 0.4 |
| WT3 | 1.9 ± 0.2 | 2.9 ± 0.2 | 3.2 ± 0.3 |
| WT4 | 3.2 ± 0.3 | 4.8 ± 0.5 | 4.8 ± 0.5 |
| WT5 | 1.8 ± 0.2 | 2.5 ± 0.2 | 2.9 ± 0.3 |
| WT6 | 2.9 ± 0.3 | 3.7 ± 0.4 | 3.9 ± 0.4 |
| WT7 | 2.9 ± 0.3 | 3.5 ± 0.3 | 3.7 ± 0.4 |
| WT8 | 0.5 ± 0.0 | 0.6 ± 0.1 | 0.6 ± 0.1 |
| WT9 | 2.3 ± 0.2 | 2.9 ± 0.3 | 3.6 ± 0.3 |
| MDR1 | 1.1 ± 0.1 | 1.5 ± 0.1 | 2.1 ± 0.2 |
| MDR2 | 2.0 ± 0.2 | 2.6 ± 0.2 | 2.8 ± 0.3 |
| MDR3 | 0.6 ± 0.1 | 1.4 ± 0.1 | 1.8 ± 0.2 |
| MDR4 | 0.9 ± 0.1 | 1.2 ± 0.1 | 1.6 ± 0.1 |
| MDR5 | 0.9 ± 0.1 | 1.5 ± 0.1 | 1.8 ± 0.2 |
| MDR6 | 0.9 ± 0.1 | 1.3 ± 0.1 | 1.6 ± 0.1 |
| MDR7 | 0.5 ± 0.0 | 0.5 ± 0.0 | 0.5 ± 0.0 |
| MDR8 | 0.5 ± 0.0 | 0.6 ± 0.1 | 1.0 ± 0.1 |
| MDR9 | 0.7 ± 0.1 | 1.2 ± 0.1 | 1.8 ± 0.2 |
| PDR2 | 0.9 ± 0.1 | 2.3 ± 0.2 | 2.6 ± 0.2 |
| PDR3 | 0.6 ± 0.1 | 0.6 ± 0.1 | 0.6 ± 0.1 |
| PDR4 | 0.6 ± 0.1 | 1.3 ± 0.1 | 1.4 ± 0.1 |
| PDR5 | 2.6 ± 0.2 | 3.2 ± 0.2 | 3.7 ± 0.3 |
| PDR6 | 2.4 ± 0.2 | 3.1 ± 0.3 | 3.5 ± 0.3 |
| PDR7 | 0.5 ± 0.0 | 1.4 ± 0.1 | 2.1 ± 0.2 |
| PDR8A | 1.0 ± 0.1 | 2.1 ± 0.2 | 2.6 ± 0.2 |

| **Anoxia** | 24h (cm) | 48h (cm) | 72h (cm) |
|---|---|---|---|
| PA14 | 1.3 ± 0.1 | 1.6 ± 0.1 | 1.9 ± 0.2 |
| WT1 | 2.6 ± 0.2 | 4.5 ± 0.4 | 4.8 ± 0.5 |
| WT2 | 0.5 ± 0.0 | 0.5 ± 0.0 | 0.5 ± 0.0 |
| WT3 | 1.0 ± 0.1 | 1.6 ± 0.1 | 1.8 ± 0.1 |
| WT4 | 1.3 ± 0.1 | 1.9 ± 0.2 | 2.3 ± 0.2 |
| WT5 | 0.3 ± 0.0 | 0.3 ± 0.0 | 0.3 ± 0.0 |
| WT6 | 2.3 ± 0.1 | 3.0 ± 0.3 | 3.8 ± 0.3 |
| WT7 | 1.0 ± 0.1 | 2.5 ± 0.2 | 2.9 ± 0.3 |
| WT8 | 0.3 ± 0.0 | 0.5 ± 0.0 | 0.5 ± 0.0 |
| WT9 | 2.1 ± 0.2 | 3.5 ± 0.3 | 3.9 ± 0.4 |
| MDR1 | 0.6 ± 0.1 | 1.6 ± 0.1 | 1.8 ± 0.2 |
| MDR2 | 0.3 ± 0.0 | 0.3 ± 0.0 | 0.3 ± 0.0 |
| MDR3 | 0.6 ± 0.1 | 0.9 ± 0.1 | 0.9 ± 0.1 |
| MDR4 | 0.6 ± 0.1 | 1.9 ± 0.2 | 1.9 ± 0.2 |
| MDR5 | 0.5 ± 0.0 | 0.8 ± 0.1 | 1.2 ± 0.1 |
| MDR6 | 0.5 ± 0.0 | 0.8 ± 0.1 | 0.9 ± 0.1 |
| MDR7 | 0.4 ± 0.0 | 0.4 ± 0.0 | 0.4 ± 0.0 |
| MDR8 | 0.4 ± 0.0 | 0.4 ± 0.0 | 0.4 ± 0.0 |
| MDR9 | 0.6 ± 0.1 | 1.3 ± 0.1 | 1.5 ± 0.1 |
| PDR2 | 0.8 ± 0.1 | 1.6 ± 0.1 | 2.0 ± 0.2 |
| PDR3 | 0.5 ± 0.0 | 0.5 ± 0.0 | 0.5 ± 0.0 |
| PDR4 | 0.6 ± 0.1 | 0.6 ± 0.1 | 0.6 ± 0.1 |
| PDR5 | 2.1 ± 0.2 | 2.6 ± 0.2 | 3.2 ± 0.3 |
| PDR6 | 0.4 ± 0.0 | 0.4 ± 0.0 | 0.4 ± 0.0 |
| PDR7 | 0.3 ± 0.0 | 0.3 ± 0.0 | 0.3 ± 0.0 |
| PDR8A | 1.3 ± 0.1 | 2.6 ± 0.3 | 2.6 ± 0.3 |

Table S2: Diameters of swarming motility of WT, MDR and PRD strains art 24, 48 and 72 h in normoxia and anoxia, respectively.

| **Normoxy** | 24h (cm) | 48h (cm) | 72h (cm) |
|---|---|---|---|
| PA14 | 1.0 ± 0.1 | 1.1 ± 0.1 | 1.3 ± 0.1 |
| WT1 | 1.0 ± 0.1 | 1.3 ± 0.1 | 1.5 ± 0.2 |
| WT2 | 1.3 ± 0.1 | 1.6 ± 0.1 | 1.6 ± 0.2 |
| WT3 | 1.0 ± 0.1 | 1.3 ± 0.1 | 1.6 ± 0.1 |
| WT4 | 1.3 ± 0.1 | 1.3 ± 0.1 | 1.3 ± 0.1 |
| WT5 | 1.0 ± 0.1 | 1.1 ± 0.1 | 1.3 ± 0.1 |
| WT6 | 1.0 ± 0.1 | 1.0 ± 0.1 | 1.3 ± 0.1 |
| WT7 | 1.0 ± 0.1 | 1.1 ± 0.1 | 1.3 ± 0.1 |
| WT8 | 0.6 ± 0.1 | 0.6 ± 0.1 | 0.6 ± 0.1 |
| WT9 | 1.6 ± 0.2 | 1.6 ± 0.2 | 1.6 ± 0.2 |
| MDR1 | 1.0 ± 0.1 | 1.0 ± 0.1 | 1.1 ± 0.1 |
| MDR2 | 1.6 ± 0.1 | 1.6 ± 0.1 | 1.6 ± 0.1 |
| MDR3 | 0.6 ± 0.1 | 0.8 ± 0.1 | 1.0 ± 0.1 |
| MDR4 | 0.6 ± 0.1 | 1.0 ± 0.1 | 1.0 ± 0.1 |
| MDR5 | 0.6 ± 0.1 | 0.8 ± 0.1 | 1.0 ± 0.1 |
| MDR6 | 0.8 ± 0.1 | 1.0 ± 0.1 | 1.0 ± 0.1 |
| MDR7 | 0.6 ± 0.1 | 0.6 ± 0.1 | 0.6 ± 0.1 |
| MDR8 | 0.6 ± 0.1 | 0.6 ± 0.1 | 0.6 ± 0.1 |
| MDR9 | 0.6 ± 0.1 | 0.9 ± 0.1 | 1.5 ± 0.1 |
| PDR2 | 0.3 ± 0.0 | 0.6 ± 0.1 | 0.8 ± 0.1 |
| PDR3 | 0.6 ± 0.1 | 0.6 ± 0.1 | 0.8 ± 0.0 |
| PDR4 | 0.3 ± 0.0 | 0.3 ± 0.0 | 0.3 ± 0.0 |
| PDR5 | 1.8 ± 0.1 | 1.9 ± 0.2 | 2.0 ± 0.2 |
| PDR6 | 1.6 ± 0.1 | 1.9 ± 0.1 | 2.1 ± 0.2 |
| PDR7 | 0.6 ± 0.1 | 0.7 ± 0.1 | 0.8 ± 0.1 |
| PDR8A | 1.0 ± 0.1 | 1.0 ± 0.1 | 1.0 ± 0.1 |

| **Anoxia** | 24h (cm) | 48h (cm) | 72h (cm) |
|---|---|---|---|
| PA14 | 0.6 ± 0.1 | 0.6 ± 0.1 | 0.9 ± 0.1 |
| WT1 | 0.6 ± 0.1 | 0.7 ± 0.0 | 0.9 ± 0.1 |
| WT2 | 0.3 ± 0.0 | 0.3 ± 0.0 | 0.3 ± 0.0 |
| WT3 | 0.6 ± 0.1 | 1.0 ± 0.1 | 1.3 ± 0.1 |
| WT4 | 0.6 ± 0.1 | 0.6 ± 0.1 | 0.6 ± 0.1 |
| WT5 | 0.6 ± 0.1 | 0.6 ± 0.1 | 0.6 ± 0.1 |
| WT6 | 0.8 ± 0.1 | 1.3 ± 0.1 | 1.3 ± 0.1 |
| WT7 | 0.6 ± 0.1 | 0.6 ± 0.1 | 0.8 ± 0.1 |
| WT8 | 0.3 ± 0.0 | 0.5 ± 0.0 | 0.5 ± 0.0 |
| WT9 | 0.8 ± 0.1 | 0.8 ± 0.1 | 0.8 ± 0.1 |
| MDR1 | 0.6 ± 0.1 | 0.6 ± 0.1 | 0.6 ± 0.1 |
| MDR2 | 0.3 ± 0.0 | 0.5 ± 0.0 | 0.5 ± 0.0 |
| MDR3 | 0.6 ± 0.1 | 0.6 ± 0.1 | 0.6 ± 0.1 |
| MDR4 | 0.8 ± 0.1 | 0.8 ± 0.1 | 0.8 ± 0.1 |
| MDR5 | 0.6 ± 0.1 | 0.6 ± 0.1 | 0.6 ± 0.1 |
| MDR6 | 0.6 ± 0.1 | 0.6 ± 0.1 | 0.6 ± 0.1 |
| MDR7 | 0.5 ± 0.0 | 0.5 ± 0.0 | 0.5 ± 0.0 |
| MDR8 | 0.6 ± 0.1 | 0.6 ± 0.1 | 0.6 ± 0.1 |
| MDR9 | 0.6 ± 0.1 | 0.6 ± 0.1 | 0.6 ± 0.1 |
| PDR2 | 0.3 ± 0.0 | 0.6 ± 0.1 | 0.6 ± 0.1 |
| PDR3 | 0.5 ± 0.0 | 0.5 ± 0.0 | 0.5 ± 0.0 |
| PDR4 | 0.3 ± 0.0 | 0.3 ± 0.0 | 0.3 ± 0.0 |
| PDR5 | 0.6 ± 0.1 | 0.8 ± 0.1 | 1.0 ± 0.1 |
| PDR6 | 0.4 ± 0.0 | 0.5 ± 0.0 | 0.6 ± 0.1 |
| PDR7 | 0.3 ± 0.0 | 0.3 ± 0.0 | 0.3 ± 0.0 |
| PDR8A | 0.8 ± 0.1 | 0.9 ± 0.1 | 1.0 ± 0.1 |
